# Mitochondrial Dynamics Disorder Drives Nucleus Pulposus Cell Senescence in Lumbar Scoliosis of Aging Bipedal Rats Under Asymmetric Force

**DOI:** 10.1155/sci/1581661

**Published:** 2026-04-06

**Authors:** Ji Wang, Daigui Cao, Jing Peng, Xu Zhou, Zhiwei Liu, Xinxing Wang, Anwei Zhang, Kai Shen

**Affiliations:** ^1^ Department of Orthopedics, Chongqing General Hospital, Chongqing University, Chongqing, 401147, China, cqu.edu.cn

**Keywords:** asymmetric force, degenerative lumbar scoliosis, mitochondrial dynamics, nucleus pulposus cell senescence

## Abstract

**Objective:**

The purpose of this study was to investigate the effect of mitochondrial dynamics disorder driving nucleus pulposus cell (NPC) senescence in lumbar scoliosis of aging bipedal rats under asymmetric force.

**Methods:**

A rat model of lumbar scoliosis with asymmetric force was established using nickel–titanium springs and anchorage screws. The condition of lumbar scoliosis was observed by X‐ray. MicroCT was used for 3D reconstruction of the microporous structure of the lumbar endplates. Histopathological changes in the L4/5 intervertebral disc were observed using Hematoxylin and Eosin (H&E) staining and Safranin O‐Fast Green staining. Immunohistochemical staining was used to observe the expression of IL‐8, IL‐6, MMP‐3, and MMP‐13 in the cells of the L4/5 intervertebral disc tissue. Western blot was performed to analyze the protein expression of p53, p21, p16, p‐p65, NLRP3, mitofusin 2 (Mfn2), dynamin‐related protein 1 (Drp1), OPA1, and Fis1. The mitochondrial morphology in NPCs of the L4/5 intervertebral disc was observed by transmission electron microscopy (TEM). The levels of superoxide dismutase (SOD), malondialdehyde (MDA), and adenosine triphosphate (ATP) in the nucleus pulposus tissue of lumbar scoliosis were measured using commercial assay kits. Reactive oxygen species (ROS) content in the nucleus pulposus tissue was quantified by flow cytometry.

**Results:**

X‐ray and MicroCT‐3D revealed that rats in the asymmetric group exhibited significant scoliosis deformity, accompanied by marked reductions in bone mineral density (BMD), tissue mineral density (TMD), bone volume fraction (BV/TV), and trabecular thickness (Tb.Th). H&E staining and Safranin O‐Fast Green staining demonstrated that asymmetric force significantly exacerbated pathological changes in the intervertebral disc, particularly damage to the cartilage endplate, chondrocyte necrosis, and hyperplasia. Immunohistochemical results indicated a significant increase in the positive expression of IL‐8, IL‐6, MMP‐3, and MMP‐13 in the asymmetric group, suggesting a synergistic effect of aging and asymmetric force in amplifying inflammatory responses and matrix degradation. Western blot analysis showed that the expression of senescence‐associated proteins (p53, p21, and p16) and inflammation‐related proteins (p‐p65, NLRP3) was significantly upregulated in the asymmetric group, indicating that asymmetric force accelerated the senescence of NPCs and activated inflammatory pathways. TEM revealed that asymmetric force markedly aggravated mitochondrial swelling and structural damage in the L4/5 intervertebral disc, with the most severe mitochondrial injury observed in the 48‐week asymmetric group. Western blot further demonstrated that the expression of mitochondrial fusion proteins (Mfn2, OPA1) was significantly decreased, while mitochondrial fission proteins (Drp1, Fis1) were significantly increased in the asymmetric group, indicating disrupted mitochondrial fission–fusion balance. Additionally, the asymmetric loading group exhibited significantly reduced SOD activity and ATP levels, along with elevated MDA content, suggesting oxidative stress‐induced mitochondrial dysfunction. Flow cytometry results confirmed a significant increase in ROS levels in the asymmetric group, highlighting that asymmetric force intensified oxidative stress in the nucleus pulposus tissue, and this effect was further exacerbated with aging.

**Conclusion:**

Asymmetric force drives the NPC senescence by disrupting mitochondrial fission–fusion balance, triggering oxidative stress and inflammatory responses, ultimately leading to the progression of degenerative lumbar scoliosis. This pathological cascade is significantly exacerbated in the context of aging.

## 1. Introduction

Intervertebral disc degeneration (IVDD), as the core pathological process of spinal degenerative diseases, is characterized by structural degeneration and functional impairment caused by the dysregulation of microenvironmental homeostasis within the disc [1]. A key pathological hallmark of IVDD is the senescence and reduced viability of disc cells, particularly nucleus pulposus cells (NPCs), which are closely associated with mitochondrial dysfunction [2]. The intervertebral disc comprises an outer annulus fibrosus and an inner nucleus pulposus [3–5]. With aging, metabolic dysregulation of the extracellular matrix, loss of hydration in the nucleus pulposus, and structural disruption of the annulus fibrosus collectively lead to biomechanical deterioration of the disc, ultimately contributing to the pathogenesis of degenerative lumbar scoliosis (DLS). DLS, a common three‐dimensional spinal deformity in middle‐aged and elderly populations, is typically characterized by lateral curvature of the lumbar spine with vertebral rotation, often accompanied by spinal stenosis, neural compression, and chronic low back pain, severely impairing patients’ quality of life [6, 7].

Studies have demonstrated that asymmetric mechanical loading on the spine in the upright human posture is a critical factor driving scoliosis progression [8]. This aberrant mechanical loading not only induces fatigue damage to disc tissues but also triggers a vicious cycle of “mechanical stress‐cellular damage‐functional decline” through the activation of oxidative stress and inflammatory cascades. Mitochondria, as central organelles governing cellular energy metabolism and apoptosis regulation, exhibit significant alterations in their dynamics (fission/fusion balance) during IVDD. Specifically, degenerated discs display a characteristic shift toward enhanced mitochondrial fragmentation with suppressed fusion, accompanied by collapse of mitochondrial membrane potential and impaired ATP synthesis [9]. Such dynamic imbalance is recognized as a hallmark of NPC senescence. Investigating the interplay between mitochondrial dynamics dysfunction and NPC senescence holds significant implications for elucidating the pathogenesis of DLS.

This study aims to investigate how mitochondrial dynamics dysfunction under asymmetric force drives NPC senescence and further contributes to the progression of lumbar scoliosis. By establishing an aging bipedal rat model, we will examine alterations in mitochondrial morphology, expression of fission/fusion‐related proteins, oxidative stress levels, and inflammatory cytokine profiles within NPCs. These findings are expected to provide a mechanistic foundation for developing targeted therapeutic strategies against DLS.

## 2. Materials and Methods

### 2.1. Animals

Thirty‐six female Sprague‐Dawley (SD) rats (SPF grade, body weight 220–250 g) were purchased from Chengdu Dashuo Biological Co., Ltd. The rats were housed under controlled conditions with a temperature of 22–24°C, humidity of 50%–60%, and a 12 h light/dark cycle, with free access to food and water. The animal experimental protocol for this study was approved by the Animal Ethics Committee of Chongqing People’s Hospital.

### 2.2. Lumbar Scoliosis of Aging Bipedal Rats Under Asymmetric Force

Rats were anesthetized via intraperitoneal injection of 2% pentobarbital sodium (40 mg/kg) and positioned supine on the operating table. Bilateral axillary incisions were made to bluntly dissect and transect the humeral heads along with surrounding muscles and nerves, followed by ligation of the brachial artery and vein. The tail base was suture‐ligated, and the distal tail was amputated with surgical scissors. Postoperative bipedal rats were carefully housed, with gradual elevation of food placement to enforce a standing posture for feeding, thereby training upright activity.

After 16 weeks of adaptation to bipedal standing (skeletal maturity stage), rats underwent fasting for 12 h preoperatively. The rats were injected with penicillin (100,000 U/kg) intraperitoneally before surgery to prevent infection. Under pentobarbital anesthesia, the L1–L6 spinal segment was identified, and a midline incision was made after standard disinfection. The right paraspinal muscles at L1–L6 were dissected using microsurgical tools to expose vertebral structures. Anchor insertion points were marked at the midpoint of the L1 and L6 transverse processes. A 25‐gauge needle was angled 30–40° anteroinferiorly into the vertebral body (depth: 5 mm), with intraoperative X‐ray confirmation of positioning. Self‐tapping titanium alloy anchorage screws (1.2 × 6.0 mm) were then implanted. A prestretched nitinol spring (Ultra Light 0–100 g) was fixed between L1 and L6 screws using nonabsorbable sutures, generating asymmetric force on the right lumbar spine. The wound was irrigated and closed in layers, and rats were fed for 24, 36, or 48 weeks postoperatively.

Rats were divided into six groups (*n* = 6/group): 24‐week control group, 24‐week asymmetric group, 36‐week control group, 36‐week asymmetric group, 48‐week control group, and 48‐week asymmetric group.

### 2.3. X‐Ray Examination

At designated time points, anteroposterior and lateral lumbar spine X‐ray imaging was performed. Rats were anesthetized via intraperitoneal injection of 2% pentobarbital sodium and positioned on the X‐ray table. Imaging parameters included a tube‐to‐table distance of 150 cm, voltage of 75 kV, and current of 16 mAs. Cobb angles were quantified using ImageJ software (NIH, USA) to confirm DLS progression. Lateral lumbar X‐ray images were additionally acquired using a digital radiography (DR) system (Ultimax, TOSHIBA, Japan) for structural assessment.

### 2.4. MicroCT‐3D

Rats were euthanized via pentobarbital sodium overdose. The lumbar spine (L1–L6) was dissected and isolated, with surrounding soft tissues removed while preserving bony structure, intervertebral disc, and facet joint. Specimens were scanned using a MicroCT system (Skyscan 1076, Belgium). Before scanning, specimens were aligned to ensure parallel orientation between vertebral endplates and the scanning plane.

Set MicroCT scanning parameters: voltage 70 kV, current 141 μA, pixel size 9.485 μm, rotational scan range 180.0°, angular increment 0.4°. After scanning, import all images into the NRecon software for reconstruction to obtain cross‐sectional images of L1‐L6 vertebrae in rat spine specimens. Since degeneration is relatively prominent in intervertebral spaces adjacent to the apical vertebra of scoliosis, reconstructed images of the inferior endplate of the L3 vertebra located at the apex of spinal curvature were selected for study.

Translate the selected L3 vertebral inferior endplate cross‐sectional images into Mimics software. Adjust appropriate bone grayscale thresholds to reconstruct 3D models of the endplate and adjacent vertebral bone. Bony endplate regions from different scan layers were selected as regions of interest (ROI). To reconstruct canal structures within the endplate, osseous components within the ROI were selectively removed. Subsequently, 3D modeling was performed on the remaining canal structures within the endplate, ultimately generating a three‐dimensional reconstruction map of the intra‐endplate canal network.

Bone volume fraction (BV/TV) is defined as the ratio of bone volume to total specimen volume. The L3 inferior endplate 3D model reconstructed from MicroCT was divided into convex and concave sides along the longitudinal axis of the endplate. CTAn software was applied to calculate the bone volume fraction of the convex and concave sides of the L3 vertebral inferior endplate. Using Mimics software, a coronal plane image passing through the midpoint of the endplate’s longitudinal axis was selected. The measurement region on the concave side endplate was first defined, with its length set as 100%. The thickness of the endplate at 25%, 50%, and 75% positions was measured, and the average value was taken to represent the endplate thickness in the concave region. Subsequently, the corresponding convex side endplate was selected on the coronal image for thickness measurement, and the convex region endplate thickness was determined using the same method. Endplate bone volume fraction = Bone volume of L3 inferior endplate/Total volume of the L1‐L6 spine specimen.

The measurement protocol was designed to specifically avoid vascular pores and heterogeneous regions: Using Mimics software, we first generated a 3D grayscale map based on bone mineral density thresholds (BMD; ≥600 mg HA/cm^3^) to isolate osseous tissue from vascular channels. The coronal plane bisecting the endplate’s longitudinal midpoint was then selected, and measurement points at 25%, 50%, and 75% were positioned only within continuous trabecular regions identified by CTAn software as having uniform density (coefficient of variation <5% within a 0.5 mm^3^ voxel neighborhood). Any points falling within low‐density voids (<400 mg HA/cm^3^) or areas with standard deviation >15% were automatically excluded and repositioned adjacent to the nearest homogeneous region. This ensured thickness measurements captured true endplate bone density without vascular pore interference.

### 2.5. H&E Staining and Safranin O/Fast Green Staining

The L4/5 intervertebral disc tissues were fixed in 4% paraformaldehyde buffer for 48 h, embedded in paraffin, and sectioned into 5 μm slices. The sections were treated with hematoxylin (H9627, Sigma Aldrich) and eosin solution (YE2080, Hefei Bomei Biotechnology Co., Ltd.) and safranin O‐fast green cartilage staining kit (G1371, Solarbao Life Science Co., Ltd., Beijing, China) after dewaxing and hydration, adhering to the manufacturer’s instructions. The sections were mounted with neutral gum, and the pathomorphological changes of bone tissue were observed using the BA210Digital optical microscope.

### 2.6. Immunohistochemistry

The nucleus pulposus tissues of lumbar scoliosis were fixed in 4% paraformaldehyde, dehydrated, cleared with dimethylbenzene, and then embedded in paraffin, after which they were sectioned into 4 μm sections. The sections were dewaxed and hydrated, soaked in 3% citric acid solution for 40 min for antigen repair, and incubated with 3% H_2_O_2_ for 10 min to block the activity of endogenous peroxidase. After blocking in goat serum for 20 min at room temperature, the sections were incubated with primary antibodies against MMP‐3 (18165‐1‐AP, Proteintech), MMP‐13 (17873‐1‐AP, Proteintech), IL‐6 (GB11117, Servicebio), and IL‐8 (bs‐0780R, Bioss) at 4°C overnight. The next day, goat anti‐rabbit labeled with HRP (GB22303, Servicebio) was added and incubated at room temperature for 50 min, followed by DAB color (ZLI‐9018, ZSGB‐BIO, China) development, hematoxylin (LM10N13, Beijing Bailingwei Technology Co., Ltd., China) restaining, and dehydrated, transparent, and neutral gum sealing tablets. The images were taken using an optical microscope (BA400Digital, Mike Audi Industrial Group Co., Ltd., Guangdong, China). The positive regions were measured with ImageJ.

### 2.7. Assay of SOD, MDA, and ATP Levels

The nucleus pulposus tissue from a scoliotic lumbar intervertebral disc was collected, ground into the tissue homogenate, and centrifuged at 3000 rpm for 15 min to obtain the supernatant. Commercial assay kits for superoxide dismutase (SOD; A001‐3‐1), malondialdehyde (MDA; A003‐1‐1), and adenosine triphosphate (ATP; A095‐1‐1) were purchased from the Nanjing Jiancheng Bioengineering Institute (Jiangsu, China). Following the manufacturer’s protocols, SOD activity was measured using the WST‐1 colorimetric method. MDA content was determined via the thiobarbituric acid (TBA) method. ATP levels were quantified using the phosphomolybdic acid colorimetric method.

### 2.8. Western Blot

Total protein was extracted from the lumbar nucleus pulposus tissue with RIPA lysis lysate. The BCA kit (P0009, Beyotime Biotechnology) was used to determine the protein concentration and prepare the protein sample. The protein was denatured by heating at 95°C for 5 min. The 20 μg protein samples were separated by electrophoresis on SDS‐PAGE gel and transferred to the PVDF membrane. The samples were enclosed in skim milk powder at room temperature for 1 h, and added with primary antibody anti‐p53 (A0263, abclonal, 1:2000), anti‐p21 (A1483, abclonal, 1:1000), anti‐p16 (A23882, abclonal, 1:1000), anti‐p‐p65 (AP0475, abclonal, 1:1000), anti‐NLRP3 (A24294, abclonal, 1:1000), anti‐Mfn2 (A19678, abclonal, 1:2000), anti‐Drp1 (A2586, abclonal, 1:2000), anti‐OPA1 (DF8587, abclonal, 1:2000), and anti‐Fis1 (A19666, abclonal, 1:1000) and incubated at 4°C overnight. After TBST washing for 3 times, Goat anti‐Rabbit IgG (H + L) HRP (S0001, affbiotech, 1:5000) secondary antibody was added, incubated at room temperature for 1 h, and then washed with TBST for 3 times. Following the membrane wash, the ECL Hypersensitive Chemiluminescence kit (17046, Zen‐Bio) was used to develop color, which was then exposed and processed in a dark room. β‐actin (AC026, abclonal) was used as the internal control. Using an Amersham Imager 600, the bands were visualized, and the density of the protein bands was measured with ImageJ software.

### 2.9. Transmission Electron Microscope (TEM)

The nucleus pulposus tissue of the L4/5 intervertebral disc was fixed with 2% glutaraldehyde at 4°C overnight and then washed three times with PBS. It was fixed with 1% osmium tetroxide for 2 h and washed three times with PBS. Dehydration was carried out with gradient acetone (30%→50%→70%→80%→90%→95%→100%→100%→100%), embedded with Epon812, and prepared into ultrathin sections. It was stained with uranyl acetate for 10 min, rinsed with running water for 2 min, stained with lead citrate for 2 min, and rinsed with running water for 2 min. The morphological changes of mitochondria were observed, and images were collected using a JEM‐1400FLASH transmission electron microscope (JEOL).

### 2.10. Flow Cytometry

Take the nucleus pulposus tissue, cut it into pieces with surgical scissors, centrifuge it, and wash it twice with PBS at 2500 *r*/min for 6 min. Discard the supernatant, digest it, and repeat the process once. Transfer the cleaned medullary tissue to a 1.5 mL centrifuge tube and cut it as much as possible with sterile scissors. Add an appropriate amount of type II collagenase (concentration usually 0.2%−0.4%, prepared in PBS) and ensure that the tissue is completely macerated. The centrifuge tubes were placed in a shaker at 37°C and digested by shaking at low speed for 15 min, and the digestion was terminated with complete medium. Resuspend the precipitate with PBS to prepare a single‐cell suspension. Collect the above cells and adjust the cell concentration to 1 × 10^6^ cells/mL. Add DCFH‐DA with a concentration of 10 μmol/L [10], and incubate at 37°C for 20 min. Mix it every 5 min to ensure full interaction between the probe and the cells. Set unstained cells as the negative cell control. Wash with PBS to remove the DCFH‐DA that has not entered the cells, and detect the ROS level using a flow cytometer (Cytoflex, Beckman). Analyze the flow cytometry data with the software CytExpert and export the results.

### 2.11. Statistical Analysis

The SPSS 22.0 software was applied for data processing and statistical analysis. The experimental data were all expressed as mean ± standard deviation (SD). Data normality was assessed using the Shapiro–Wilk test, and homogeneity of variances was verified using Levene’s test. An independent samples *t*‐test was used for the comparison between two groups, and a one‐way analysis of variance (one‐way ANOVA) was used for the comparison among multiple groups. For multiple comparisons following a significant one‐way ANOVA, Tukey’s post‐hoc test was applied to control the family‐wise error rate. A *p*‐value < 0.05 was considered to indicate a statistically significant difference.

## 3. Results

### 3.1. The Condition of Lumbar Scoliosis of Aging Bipedal Rats Under Asymmetric Force

As shown in the X‐ray images (Figure 1A), in both the anteroposterior and lateral views, the lumbar vertebrae of the rats in the asymmetric force group (at 24 weeks, 36 weeks, and 48 weeks) showed obvious scoliosis deformities, while no obvious scoliosis was observed in the lumbar vertebrae of the rats in the control group (at 24 weeks, 36 weeks, and 48 weeks). As shown by MicroCT‐3D (Figure 1B), compared with the 24‐week asymmetric group, the BMD and tissue mineral density (TMD) of the 48‐week asymmetric group were significantly decreased, indicating that with the increase of age and the continuous action of asymmetric force, the degree of mineralization of bone tissue gradually decreases. When comparing the asymmetric force group at 48 weeks with the control group at 48 weeks, the BMD of the asymmetric force group was significantly lower, suggesting that the asymmetric force significantly affects the BMD and accelerates the process of osteoporosis. Compared with the control group (at 24 weeks, 36 weeks, and 48 weeks), the BV/TV of the asymmetric force group (at 24 weeks, 36 weeks, and 48 weeks) was significantly reduced, indicating that under the action of asymmetric force, the proportion of bone volume relative to the total volume decreases, suggesting the rarefaction of the trabecular bone structure. When comparing the asymmetric force group at 48 weeks with the asymmetric group at 24 weeks, the trabecular thickness (Tb.Th) of the 48‐week group was significantly lower, indicating that with the increase of age, the trabecular bone becomes thinner and the mechanical strength of the bone tissue decreases. When comparing the asymmetric force group at 48 weeks with the asymmetric force group at 36 weeks, the Tb.Th of the 48‐week group was significantly lower, indicating that the thickness of the trabecular bone further decreases under the continuous action of asymmetric force. There were no significant differences in trabecular number (Tb.N), trabecular separation (Tb.Sp), and structure model index (SMI) among the groups, indicating that the asymmetric force has a relatively small impact on the number, spacing, and structural model of the trabecular bone.

Figure 1The condition of lumbar scoliosis of aging bipedal rats under asymmetric force. (A) The condition of lumbar scoliosis was observed by X‐ray. (B) MicroCT was used for 3D reconstruction of the microporous structure of the lumbar endplates.  ^∗^ *p* < 0.05,  ^∗∗^ *p* < 0.01, and  ^∗∗∗^ *p* < 0.001.

**Figure:**
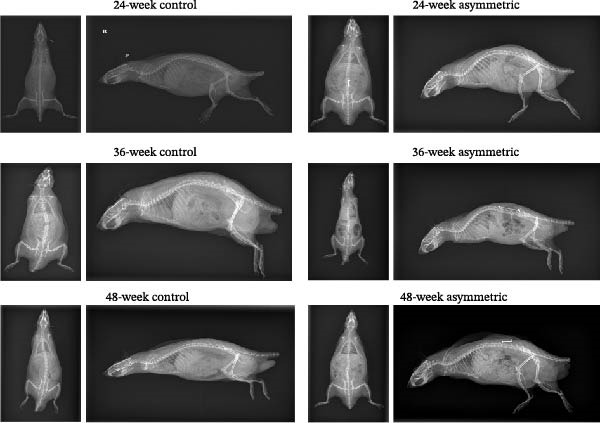
(A)

**Figure:**
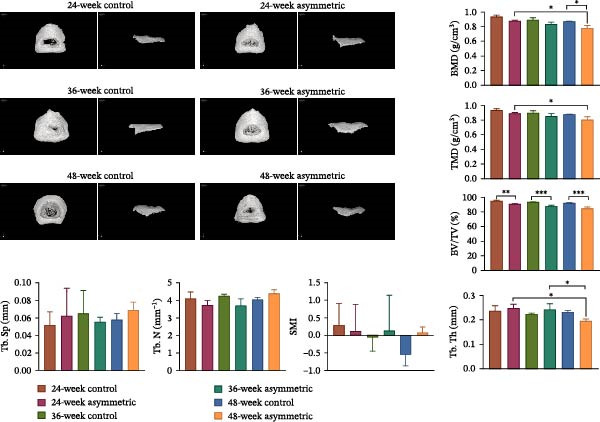
(B)

### 3.2. Pathological Changes of Intervertebral Disc Tissue in Aging Bipedal Rats

The results of H&E staining (Figure 2A) showed that the asymmetric force significantly aggravated the pathological changes of the intervertebral disc, especially the damage to the cartilaginous endplate. With the increase in age (48 weeks), the degree of the lesion became more severe, manifested as necrosis, degeneration, and hyperplasia of chondrocytes. This suggests that the asymmetric force and age are important factors in the degeneration of the intervertebral disc. There were no obvious pathological changes observed in the annulus fibrosus and the nucleus pulposus, indicating that their structures were relatively stable. The results of Safranin O‐fast green staining (Figure 2B) showed that the asymmetric force significantly aggravated the hyperplasia and thickening of the cartilaginous endplate and chondrocytes, especially in the asymmetric force group at 48 weeks, which showed a significant compensatory response. With the increase of age, the changes in the cartilaginous endplate and chondrocytes in the asymmetric force group were more remarkable, indicating that the synergistic effect of age and asymmetric force aggravated the degeneration of the intervertebral disc.

Figure 2Pathological changes of intervertebral disc tissue in aging bipedal rats. (A) Histopathological changes in the L4/5 intervertebral disc were observed using H&E staining. Green arrow: chondrocyte degeneration and necrosis. Scale bar: 50 μm. (B) Histopathological changes in the L4/5 intervertebral disc were observed using Safranin O‐Fast Green staining. Red arrow: chondrocyte proliferation. Scale bar: 50 μm.  ^∗^ *p* < 0.05,  ^∗∗^ *p* < 0.01, and  ^∗∗∗^ *p* < 0.001.

**Figure:**
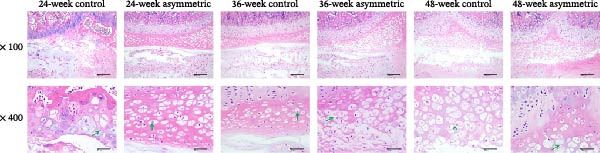
(A)

**Figure:**
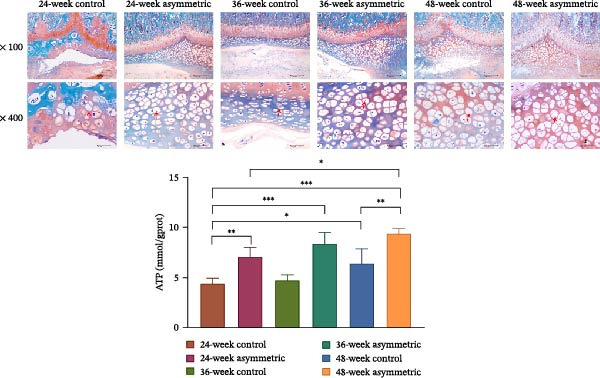
(B)

### 3.3. NPC Senescence and Inflammatory Factors in Aging Bipedal Rats

The results of immunohistochemistry (Figure 3A) showed that compared with the control group (at 24 weeks, 36 weeks, and 48 weeks), the positive expressions of IL‐8, IL‐6, MMP‐3, and MMP‐13 in the asymmetric force group (at 24 weeks, 36 weeks, and 48 weeks) significantly increased. With the increase of age, the expressions of IL‐8, IL‐6, MMP‐3, and MMP‐13 in the asymmetric force group significantly increased. This indicates that the synergistic effect of age and asymmetric force aggravates the inflammatory response and matrix degradation. The results of the Western blot (Figure 3B) showed that compared with the control group (at 24 weeks, 36 weeks, and 48 weeks), the expressions of senescence‐related proteins (p53, p21, and p16) and inflammation‐related proteins (p‐p65, NLRP3) in the asymmetric force group (at 24 weeks, 36 weeks, and 48 weeks) were significantly upregulated, indicating that the asymmetric force accelerates the senescence process of NPCs and activates the inflammatory response. With the increase in age (at 48 weeks), the expressions of p53, p21, p16, p‐p65, and NLRP3 further increased.

Figure 3NPC senescence and inflammatory factors in aging bipedal rats. (A) Immunohistochemical staining was used to observe the expression of IL‐8, IL‐6, MMP‐3, and MMP‐13 in the cells of the L4/5 intervertebral disc tissue. Scale bar : 50 μm, original magnification: ×400. (B) Western blot was performed to analyze the protein expression of p53, p21, p16, p‐p65, and NLRP3.  ^∗^ *p* < 0.05 and  ^∗∗^ *p* < 0.01.

**Figure:**
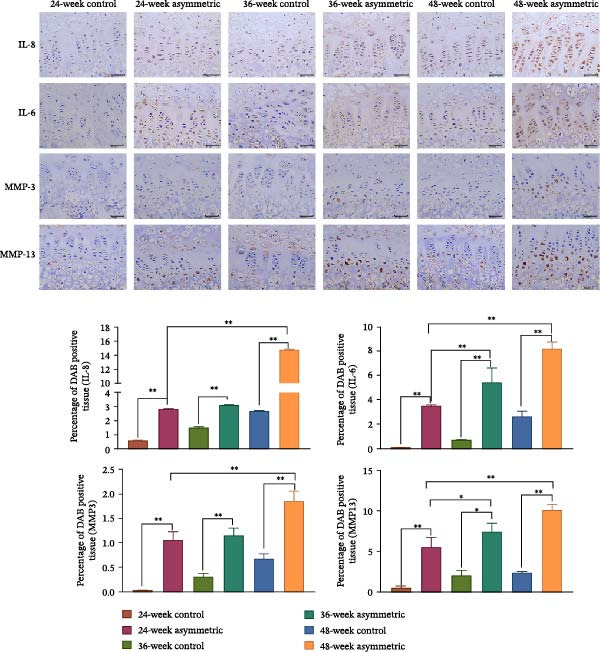
(A)

**Figure:**
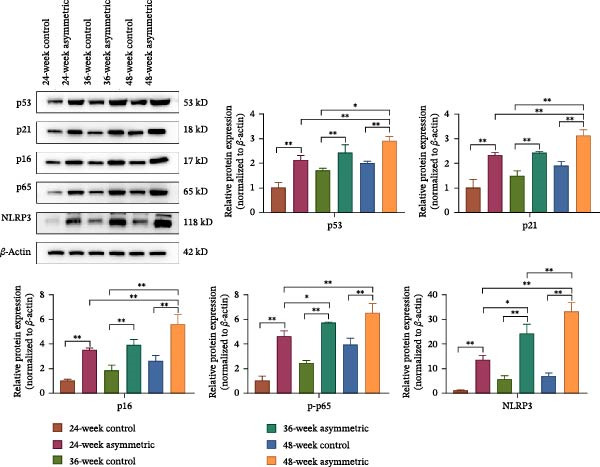
(B)

### 3.4. Mitochondrial Morphology and Mitochondrial Dynamics in Aging Bipedal Rats

The results of transmission electron microscopy (TEM) (Figure 4A) showed that the asymmetric force significantly aggravated the swelling and structural damage of mitochondria in the L4/5 intervertebral disc, especially in the asymmetric force group at 48 weeks, where the mitochondrial damage was the most severe. And this damage became more severe with the increase in age. The results of the Western blot (Figure 4B) showed that compared with the control group (at 24 weeks, 36 weeks, and 48 weeks), the expressions of mitochondrial fusion proteins Mfn2 and OPA1 in the asymmetric force group (at 24 weeks, 36 weeks, and 48 weeks) were significantly decreased, while the expressions of mitochondrial fission proteins Drp1 and Fis1 were significantly increased. This indicates that the asymmetric force significantly disrupted the balance between mitochondrial fission and fusion, inhibited the mitochondrial fusion process, and activated the mitochondrial fission process. This imbalance in mitochondrial dynamics may lead to mitochondrial dysfunction and further exacerbate DLS.

Figure 4Mitochondrial morphology and mitochondrial dynamics in aging bipedal rats. (A) The mitochondrial morphology in NPCs of the L4/5 intervertebral disc was observed by TEM. Original magnification: ×50000, nucleus (N), mitochondria (Mi), rough endoplasmic reticulum (RER), and slight swelling of mitochondria (red arrows). (B) Western blot was performed to analyze the protein expression of Mfn2, Drp1, OPA1, and Fis1.  ^∗^ *p* < 0.05 and  ^∗∗^ *p* < 0.01.

**Figure:**
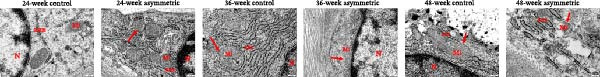
(A)

**Figure:**
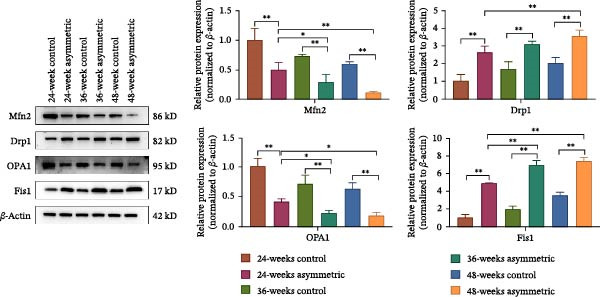
(B)

### 3.5. Oxidative Stress of Lumbar Nucleus Pulposus in Aging Bipedal Rats

The results showed (Figure 5A) that compared with the control group (at 24 weeks, 36 weeks, and 48 weeks), the activity of SOD and the level of ATP in the asymmetric force group (at 24 weeks, 36 weeks, and 48 weeks) were significantly decreased, while the content of MDA was significantly increased. With the increase of age, the activity of SOD and the level of ATP both showed a downward trend in both the control group and the asymmetric force group, and the content of MDA showed an upward trend in both the control group and the asymmetric force group. The results of flow cytometry (Figure 5B) showed that compared with the control group (at 24 weeks, 36 weeks, and 48 weeks), the content of reactive oxygen species (ROS) in the asymmetric force group (at 24 weeks, 36 weeks, and 48 weeks) was significantly increased, indicating that the asymmetric force significantly increased the level of oxidative stress in the nucleus pulposus tissue. And this effect became more pronounced with the increase of age.

Figure 5Oxidative stress of lumbar nucleus pulposus in aging bipedal rats. (A) The levels of SOD, MDA, and ATP in the nucleus pulposus tissue of lumbar scoliosis were measured using commercial assay kits. (B) ROS content in the nucleus pulposus tissue was quantified by flow cytometry.  ^∗^ *p* < 0.05 and  ^∗∗^ *p* < 0.01.

**Figure:**
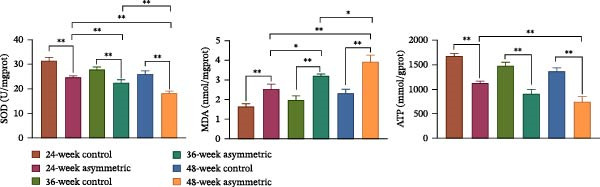
(A)

**Figure:**
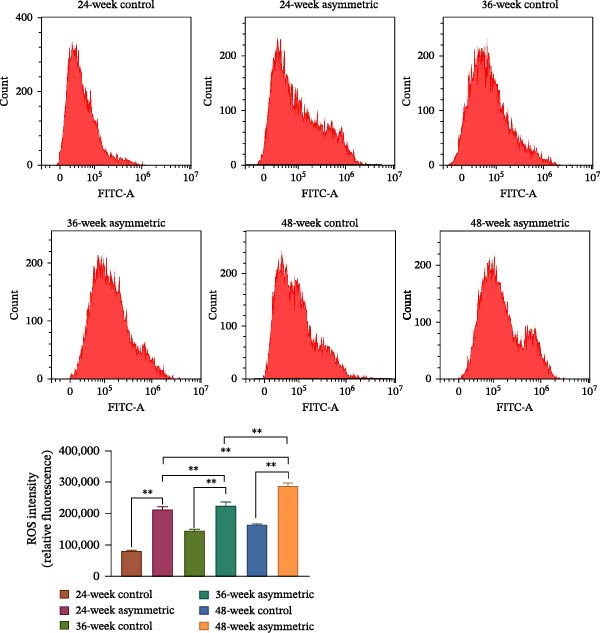
(B)

## 4. Discussion

The intervertebral disc is the largest avascular tissue in the human body and plays a crucial role in maintaining the structure and function of the spine. Previous studies have shown that among the surrounding structures of the intervertebral disc, the vertebral endplate is the main source of nutrients for the intervertebral disc [11]. There is a close internal connection between the pathological changes of the vertebral endplate and the degenerative process of the intervertebral disc [12]. In this study, under the action of asymmetric force, the lumbar endplates of aging bipedal rats showed a significant decrease in BMD, TMD, and Tb.Th became thinner. These changes indicate that the microporous structure of the endplate has undergone significant degeneration, and the trabecular bone network structure has become sparse and fragile, thereby weakening the mechanical support function of the endplate. These results further support the promoting effect of asymmetric force on the degeneration of the lumbar endplate and the intervertebral disc. The histopathological results further confirm that the combination of asymmetric force and aging has aggravated the pathological changes of the intervertebral disc tissue in aging bipedal rats, especially the damage to the cartilaginous endplate and the abnormal hyperplasia of chondrocytes. These factors all impair the normal physiological function of the intervertebral disc.

A close interplay exists between nucleus pulposus (NP) cell senescence and inflammatory responses [13]. Senescent NP cells secrete a spectrum of senescence‐associated secretory phenotype (SASP) factors, including multiple pro‐inflammatory cytokines, which activate inflammatory responses in neighboring cells [14]. Conversely, the inflammatory microenvironment further accelerates cellular senescence. P53, a critical tumor suppressor, is activated under cellular stress and regulates downstream genes to induce cell cycle arrest, senescence, or apoptosis. P21, a downstream target of p53, inhibits cyclin‐dependent kinases (CDKs), leading to cell cycle arrest and promoting senescence [15]. P16 also participates in senescence regulation, and its overexpression suppresses cell proliferation, driving cells into a senescent state. These findings suggest that asymmetric mechanical loading exacerbates NPC senescence [16]. In this study, the expression of these senescence‐associated proteins further increased in the 48‐week asymmetric group with aging, indicating a synergistic effect between age and mechanical stress in accelerating NPC senescence. P‐p65, the activated form of the NF‐κB signaling pathway, acts as a central transcription factor in inflammatory responses by regulating the expression of multiple cytokines [17]. NLRP3, a core component of the inflammasome, promotes the maturation and release of IL‐1β and other cytokines upon activation, thereby amplifying inflammation [18]. Upregulation of both markers implies that asymmetric loading activates inflammatory pathways in NPCs. Notably, aging further intensified this inflammatory response, reflecting an age‐dependent enhancement of mechanical stress‐induced inflammation. Under the combined effects of asymmetric loading and aging, this mutually reinforcing relationship forms a vicious cycle, progressively disrupting the intervertebral disc microenvironment and driving the progression of lumbar scoliosis.

Mitochondrial dynamics (the dynamic balance between fission and fusion) is a central mechanism for maintaining mitochondrial function [19, 20]. Studies have confirmed that multiple proteins mediate mitochondrial fission and fusion processes [21–23]. Drp1 and Fis1 primarily regulate mitochondrial fission, while Mfn2 and OPA1 are key players in mitochondrial fusion. Excessive mechanical stress disrupts the fission/fusion equilibrium in nucleus pulposus NPCs, characterized by decreased expression of fusion‐related proteins Mfn1, Mfn2, and OPA1, and increased expression of fission‐related proteins Drp1, Mff, and Fis1, ultimately leading to mitochondrial dysfunction [24]. Madhu et al. [25] reported elevated Drp1 expression and enhanced mitochondrial fission in NP cells under hypoxic conditions. Drp1‐mediated mitochondrial fission contributes to oxidative stress‐induced apoptosis in annulus fibrosus cells exposed to oxidized low‐density lipoprotein [26]. Chen et al. [27] observed reduced Mfn2 expression in human degenerated intervertebral disc tissues and showed that Mfn2 overexpression alleviates oxidative stress damage in NPCs via autophagy modulation, thereby delaying IVDD. These findings collectively indicate that mechanical stress‐induced mitochondrial fission/fusion imbalance in disc cells promotes IVDD progression by regulating oxidative stress, apoptosis, and autophagy. In this study, the asymmetric mechanical loading group exhibited significantly reduced expression of Mfn2 and OPA1 and markedly increased expression of Drp1 and Fis1, suggesting activated mitochondrial fission and suppressed fusion processes. This demonstrates that asymmetric mechanical loading disrupts mitochondrial fission–fusion homeostasis.

Oxidative stress refers to a state in which intracellular ROS levels significantly exceed physiological thresholds, playing a critical role in the initiation and progression of IVDD [28]. Mitochondrial dysfunction leads to electron leakage, which promotes excessive ROS accumulation in disc cells. This elevated ROS further exacerbates mitochondrial dysfunction, creating a self‐perpetuating vicious cycle of ROS overproduction [29, 30]. Studies have demonstrated that ROS activates the NF‐κB and mitogen‐activated protein kinase (MAPK) signaling pathways, amplifying inflammatory cytokine production and accelerating apoptosis and senescence of disc cells [31]. In this study, rats subjected to asymmetric loading exhibited significantly increased ROS and MDA levels in nucleus pulposus tissue, alongside markedly reduced SOD activity and ATP levels, indicating that oxidative stress profoundly disrupts mitochondrial function. As the central organelle for cellular energy metabolism, mitochondrial dysfunction compromises ATP synthesis and weakens metabolic capacity, thereby impairing the normal physiological functions of NPCs.

The occurrence of DLS is closely related to age [32]. This study found that aging significantly exacerbates the negative effects of asymmetric force on IVDD. The 48‐week asymmetric force group exhibited more severe mitochondrial damage, elevated oxidative stress levels, and upregulated inflammatory factor expression compared to younger groups (24 weeks, 36 weeks). This may be closely associated with the decline in autophagy capacity of NPCs and the deterioration of the antioxidant defense system during aging. MicroCT‐3D results revealed that the BMD and BV/TV in the asymmetric force group significantly decreased with age, while degeneration of the endplate microporous structure impaired its nutritional supply function to the intervertebral disc, exacerbating energy metabolism disorders in NPCs. These findings suggest that age‐related degenerative background and abnormal mechanical loading exhibit synergistic effects, jointly driving the pathological progression of DLS.

This study is the first to reveal the role of mitochondrial dynamics disorder‐driven NPC senescence in an asymmetric force‐induced aging bipedal rat lumbar scoliosis model. However, there are still limitations. For instance, the specific upstream regulatory mechanisms of mitochondrial fission/fusion imbalance (such as Drp1 phosphorylation or Mfn2 ubiquitination modification) remain unclear. Additionally, whether interventions targeting mitochondrial dynamics (e.g., using MitoQ or Drp1 inhibitors) can reverse IVDD requires further validation. Future research could employ single‐cell sequencing technology to analyze the heterogeneity of senescent NPCs and explore novel therapeutic strategies targeting mitochondrial dynamics or antioxidant pathways.

## 5. Conclusion

In summary, within the aging bipedal rat model of lumbar scoliosis subjected to asymmetric force, asymmetric force disrupt mitochondrial dynamics homeostasis, induce oxidative stress and inflammatory responses, and NPC senescence, ultimately contributing to the initiation and progression of DLS. This pathological cascade is synergistically exacerbated by the aging process.

## Author Contributions

Ji Wang wrote the paper, Kai Shen applied for funds, Daigui Cao and Jing Peng designed the experiments, Xu Zhou and Zhiwei Liu finished the experiments, Xinxing Wang analyzed the data, and Anwei Zhang corrected and checked the submitted manuscript.

## Funding

This work was supported by the General Project of the Natural Science Foundation of Chongqing, China (Grant cstc2021jcyj‐msxmX0922).

## Disclosure

All authors have approved the submitted manuscript.

## Ethics Statement

All animal experiments in this study were conducted in accordance with the ethical standards for experimental animals (Ethics Committee of West China Hospital, Sichuan University, Number 20230412009).

## Conflicts of Interest

The authors declare no conflicts of interest.

## Data Availability

The data that support the findings of this study are available from the corresponding author upon reasonable request.
